# Hepatic Cirrhosis

**Published:** 1890-12-27

**Authors:** 


### THE PRACTITIONER'S MIRROR AND RETROSPECT.

[*The Editor will be glad to receive offers of co-operation and contributions from members of the profession. There is no doubt that much valuable material full of interest and instruction is at present lost to science. This is largely so in the case of* (1) *the younger and less-known members of the hospital staff* (2) *those connected with cottage hospitals, and* (3) *the great body of medical practitioners not attached to any institution. The co-operation of all is cordially invited to enable the Editor to make* THE HOSPITAL *of the utmost possible use and value to the profession. Specialists willing to review books on their special subjects are invited to communicate this fact at once. All letters should be addressed to* THE EDITOR, THE LODGE, PORCHESTER SQUARE, LONDON, W.]

### MEDICINE.

### HEPATIC CIRRHOSIS.

One of the most interesting and spirited debates in the medical section at the recent meeting of the British Medical Association at Birmingham was on Dr. Saundby's paper on the "Varieties of Hepatic Cirrhosis." The learned author distinguished no less than ten forms of cirrhosis of the liver, namely, alcoholic, or the ordinary hobnail liver; cardiac cirrhosis, from chronic venous congestion; biliary, in which jaundice is an initial symptom: the diffuse syphilitic cirrhosis; gummatous cirrhosis; tubercular; malarial; scarlatinal; rachitic; diabetic. This, however, did not seem to be sufficient. For one speaker remarked that interstitial cirrhosis had not been included; another speaker mentioned a typhoid form; a third speaker said there was a cirrhosis produced by swallowing tobacco juice; and a fourth speaker referred to acute alcoholic biliary cirrhosis. Now, we think that a mistake was made in the first instance by Dr. Saundby, who finding cirrhosis in association with various conditions, described the liver affection as a distinct condition. Cirrhosis is cirrhosis, however it is produced. And the conditions with which it is found in association may or may not be causes of the alteration of hepatic structure. Among objectors to Dr. Saundby's classification was Sir William Moore, who thought malarial cirrhosis ought not to find a place in the list. Dr. Saundby had said that malarial cirrhosis occurred after attacks of congestion of the liver consequent on ague or malarial fever. But Sir William Moore remarked that after such attacks there was usually enlargement of the liver of the fatty or lardaceous description and not cirrhosis. With regard to scarlatinal cirrhosis, the author himself confessed that it was "known only to pathologists." Of course the chief clinical interest centres in the alcoholic form of cirrhosis. But it is important to note that this may exist without causing such derangement of health as to call for medical assistance. One of the speakers observed that among the very early symptoms of cirrhosis were intermittent headaches, loss of power of concentration of the mind, and occasional numbness of the fingers and toes, or of the extremities generally. But there is another very important symptom which was especially dwelt upon by Dr. Saundby. There may be scarcely any symptoms present, and there may be no ascites, but nevertheless the patient is very liable to hæmatemesis. This is due to rupture of dilated gastric or œsophageal veins, by which the blood from the portal system reaches the superior vena cava. Such hæmatemesis is not unfrequently fatal. According to Dr. Saundby hæmatemesis is absent in all varieties of cirrhosis excepting the alcoholic variety. How this may be we confess that we do not quite understand, for whatever may cause the cirrhosis the secondary effect of the cirrhosis on neighbouring parts must be the same. Congestion of the œsophageal veins, from whatever cause produced, is very liable to lead to rupture or ulceration, and its result discharge of blood. Summing up the clinical features of alcoholic cirrhosis, the author stated that it usually occurs in the male adult; that there is a history of the abuse of alcohol; that ascites is eventually present in two-thirds of the cases; that jaundice is absent till the later stages; that hæmatemesis may occur; and that the liver is usually small. Other anatomical changes are a pale olive colour of the liver, the surface being granular, the capsule thick, the consistence tough, the bile passages nearly empty, the position of the new growth is round the cells of the acini, while newly-formed biliary canaliculi are present. To this may be added that the spleen is often enlarged. Also that those who suffer from hæmatemesis rarely suffer from abdominal dropsy, and the reverse. Dr. Saundby did not refer to melæna or hæmorrhoids as a result of cirrhosis. Neither did he mention vertigo, epistaxis, convulsions, or apoplexy, which may be sequences; the latter especially if there is an atheromatous condition of cerebral arteries, which are thereby rendered unable to bear the strain from impediment to free circulation of venous blood in the liver. Dr. Saundby considers that when ascites is present it is most desirable to attempt to get rid of it by repeated tappings, and a case of six years' standing was shown to illustrate the success of this treatment. When hæmatemesis occurs, Dr. Saundby condemns the usual treatment by styptics. Perfect quiet, and especially avoiding swallowing, are required. The action of swallowing increases the flow of blood from any rupture or ulcer of the œsophageal veins, and thus adds to the degree of hæmorrhage or hæmatemesis. This is an important modification of the plan usually adopted for hæmatemesis, and there is no doubt that it is not only novel, but also correct. Mr. Hutchinson, of Scarborough, taking part in the debate, remarked that although so many varieties of hepatic cirrhosis had been mentioned, he felt rather comforted by the consideration that none of these varieties appeared to have definite symptoms, excepting the alcoholic kind. Practising in a health resort, Dr. Hutchinson saw many cases of hepatic cirrhosis sent away for change of air, after various kinds of treatment had been tried. And not only were such cases sent to British watering-places, but they were also advised to take sea-trips, and not unfrequently they were sent abroad. After what had been advanced, Dr. Hutchinson considered the caution he was about to give would be scarcely necessary. Nevertheless, he thought that persons with cirrhosis of the liver should not be sent to sea. For persons were liable to sea-sickness, and the action of vomiting would be very likely to conduce to rupture of the dilated and congested œsophageal veins, from which, as had been demonstrated, the blood passed arises, when hæmatemesis occurs. The not uncommon termination of alcoholic cirrhosis, with symptoms of malignant jaundice, was emphasised by Dr. Saundby; and special attention was drawn to rachitic cirrhosis, which Dr. Saundby remarked is frequently supposed to be syphilitic—a view which receives some support from the benefit derived from the use of mercury in small doses. It should be mentioned that Dr. Saundby exhibited microscopic specimens of all the varieties of cirrhosis to which he referred.

